# miRNA-141 Induced Pyroptosis in Intervertebral Disk Degeneration by Targeting ROS Generation and Activating TXNIP/NLRP3 Signaling in Nucleus Pulpous Cells

**DOI:** 10.3389/fcell.2020.00871

**Published:** 2020-08-31

**Authors:** Qiaolong Xu, Hongyuan Xing, Jiaqi Wu, Weishan Chen, Ning Zhang

**Affiliations:** ^1^Department of Orthopedics, 2nd Affiliated Hospital, School of Medicine, Zhejiang University, Hangzhou, China; ^2^Department of Orthopaedics, The People’s Hospital of Cixi, Cixi, China

**Keywords:** extracellular matrix, intervertebral disk degeneration, miRNA-141, pyroptosis, TXNIP/NLRP3 signaling

## Abstract

The role and mechanism of pyroptosis in intervertebral disk (IVD) degeneration are unclear. MicroRNAs (miRNAs) regulate the viability and function of nucleus pulposus cells (NPCs) in IVDs and are related to pyroptosis. We performed microarray analyses of normal and degenerated nucleus pulposus (NP) to assess the role of pyroptosis and identify key miRNAs in IVD degeneration. We also evaluated the underlying mechanism of miRNA-mediated pyroptosis in NPCs. In addition, we demonstrated the preventative effects of miRNAs on IVD degeneration in a rat model. The levels of the pyroptosis-related proteins cleaved caspase-1, N-terminal gasdermin D (GSDMD), interleukin (IL)-1β, and IL-18 in the degenerative NP were significantly higher than those in the normal NP. miRNA-141 was significantly upregulated in the degenerated NP. miR-141 mimic suppressed the matrix synthesis function of NPCs. By contrast, reactive oxygen species (ROS) generation, and the expression of TXNIP and NLRP3 were significantly downregulated by an miR-141 inhibitor. Furthermore, the miRNA-141 inhibitor prevented the degeneration of IVDs *in vivo*. Our findings suggest that miRNA-141 induces pyroptosis and extracellular matrix (ECM) catabolism in NPCs by increasing ROS generation and activating TXNIP/NLRP3 signaling. miRNA-141-regulated pyroptosis may be a novel therapeutic target for IVD degeneration.

## Introduction

Intervertebral disk (IVD) degeneration and the consequent chronic low-back pain affect approximately 632 million people worldwide and exert social and economic effects on patients (Mathew et al., 2013). IVD degeneration imposes the highest economic burden among all musculoskeletal complaints (Manchikanti et al., 2009). The IVD, which is composed of the nucleus pulposus (NP), annulus fibrosus (AF), and cartilaginous endplates, contributes to motion, weight bearing, and flexibility while protecting the spinal cord (Bowles and Setton, 2017). The healthy NP contains normal NP cells (NPCs), which synthesize collagen II and proteoglycan. The dysfunction of NPCs and alteration of the extracellular matrix (ECM) is thought to be the origin of IVD degeneration (Kepler et al., 2013).

Many factors regulate the viability and function of NPCs. Apoptosis is one of the main causes of NPC loss and dysfunction (Song Y. et al., 2018). Apoptosis of NPCs leads to downregulation of ECM synthesis by activating the p53 signaling pathway (Jin et al., 2018). Apoptosis is also involved in several grades of IVD degeneration (Eser et al., 2017). Other factors, such as nutrients and aging, may also regulate the viability of, and ECM synthesis by NPCs (Barakat et al., 2019). In recent years, a number of studies have focused on pyroptosis, a novel inflammatory form of regulated cell death (Liu X. et al., 2016; Ruhl et al., 2018).

Canonical pyroptosis is typically mediated by active caspase-1, which cleaves the pore-forming protein gasdermin D (GSDMD). The N-terminal domain is liberated from GSDMD after cleavage and triggers pyroptosis and the release of biologically activate interleukin (IL)-1β and IL-18, generating an inflammasome-associated inflammatory response (Xue et al., 2019). Pyroptosis occurs in various types of cells and tissues. Zheng et al. (2020) reported a role for GSDME-mediated pyroptosis in cardiac injury. NLRP3-related pyroptosis is involved in the occurrence and development of diabetes mellitus and its associated complications (Yu et al., 2020). Endoplasmic reticulum-related pyroptosis plays a role in the pathogenesis of steatohepatitis. In chondrocytes, pyroptosis modulates cell viability and ECM synthesis (Hu et al., 2020). However, the role of pyroptosis in IVD degeneration is unclear. Whether pyroptosis regulates the viability of, and ECM synthesis by, NPCs needs to be investigated.

MicroRNAs (miRNAs) are small endogenous non-coding RNAs of 21–23 nucleotides that regulate gene expression (Bartel, 2009). miRNAs play critical roles in cell proliferation, function, and pyroptosis (Kong et al., 2019; Zeng et al., 2019). In IVDs, miRNAs regulate the viability and function of NPCs. miR-532 induces NPC apoptosis by downregulating Wnt/β catenin signaling (Sun et al., 2018). miR-194 the inhibits inflammatory response in NPCs by targeting TNF receptor-associated factor 6 (Kong et al., 2018). miR-7 modulates ECM degeneration of NPCs by targeting growth differentiation factor 5 (Liu W. et al., 2016). We performed microarray analyses of normal and degenerated NPs and found 108 significantly differentially expressed miRNAs (*p* < 0.01). Among them, the level of miR-141 in degenerated NP was significantly higher than that in normal NP. A prior study reported that miR-141 is related to IVD degeneration (Ji et al., 2018). Therefore, miR-141 may be a key regulator in NPCs. miR-141 modulates cell migration, proliferation, and apoptosis (Zhao et al., 2013; Li et al., 2017; Qin et al., 2019). However, whether miR-141 mediates pyroptosis in NPCs and the underlying mechanism are unclear.

We investigated the role of miR-141 in regulating pyroptosis in NPCs during IVD degeneration. We further evaluated the mechanism underlying miR-141-mediated pyroptosis in NPCs. Finally, we assessed the preventative effect of an miR-141 inhibitor on degenerated IVD in a rat model. Discovery of the effect of miR-141 on pyroptosis in NPCs may provide a novel therapeutic target for IVD degeneration.

## Materials and Methods

### Tissue Source and Cell Culture

The study was approved by the Ethics Committee of The Second Affiliated Hospital of Zhejiang University School of Medicine, and informed consent was obtained from all participating patients. Normal NPs were donated by 10 patients (10–20 years old) without a clinical history of IVD disease and degenerative NPs by 10 patients (40–50 years old) with clinical characteristics of low back pain and lower limb pain. The disk degeneration grades of the donors were evaluated according to the Pfirrmann classification by magnetic resonance imaging of the IVD. NP samples were obtained by nucleotomy and intervertebral fusion surgery under sterile conditions and processed within 1 h of being harvested.

Healthy human NPCs were purchased from Procell (Procell Life Science & Technology, Hubei, China) and cultured in Dulbecco’s modified Eagle’s medium (DMEM)-high glucose supplemented with 10% fetal bovine serum (FBS), 4 mM L-glutamine, and 1% penicillin–streptomycin at 37°C in 5% CO_2_. NPCs at passages 2–4 were harvested. We used a pH 6.8 solution adjusted with sterilized HCl (1 M) to simulate the mildly degenerated IVD and induce the degeneration of NPCs (Fu et al., 2018; Hartman et al., 2018).

### Cell Transfection

The human mimic-141 negative control (NC), miR-141 mimic, miR-141 inhibitor NC, and miR-141 inhibitor were purchased from GenePharma (Shanghai, China) and transfected according to the manufacturer’s instructions. Briefly, cells were cultured in serum-free DMEM-high glucose medium for 12 h. The miR-NC, miR-mimic, or miR-inhibitor, and Lipofectamine 2000^TM^ transfection reagent were diluted in 250 μL of Opti-MEM (Gibco, Shanghai, China). The Lipofectamine–miRNA mixture was added to the serum-free medium. Fresh medium containing 10% FBS was added to stop the transfection after 6 h.

### Cell Viability

The viability of NPCs was assessed using a Cell Counting Kit-8 (CCK8, Dojindo, Dalian, China). NPCs were seeded into a 96-well plate. At predetermined time points, the medium was removed, and the cells were treated with 10% CCK8 in 100 μL of DMEM-high glucose for 2 h at 37°C. Absorbance at 450 nm was measured using a microplate reader (Bio-Rad Laboratories, Hercules, CA, United States).

### RNA Isolation and Real-Time Quantitative PCR (RT-qPCR)

Total RNA was extracted from tissues or NPCs using RNAiso reagent (Takara, Shanghai, China) and reverse−transcribed to complementary DNA using PrimeScript^TM^ RT Master Mix or miRNA First-Strand Synthesis kits. Real-time-quantitative polymerase chain reaction (RT-qPCR) was performed using TB Green Premix Ex Taq (Takara) on an ABI StepOnePlus System (Applied Biosystems, Warrington, United Kingdom). miR-141 expression was normalized to *U6*, and those of other mRNAs to that of *18S*. The data were analyzed by the 2^(–ΔΔCT)^ method and the primers used were synthesized by Sangon Biotech (Shanghai, China; Table 1).

**TABLE 1:** Primers used in Real-time-quantitative polymerase chain reaction (RT-PCR).

Gene	Forward Primer (5Reverse Primer (5–3)
*18s*	GAATTCCCAGTAAGTGC GGGTCATA	CGAGGGCCTCACTAAAC CATC
*U6*	CTCGCTTCGGCAGCACA	AACGCTTCACGAATTTG CGT
*hsa-miR-141-3p*	GCGGCGGTAACACTGT CTGG	AACGCTTCACGAATTTG CGT
*mo-miR-141-3p*	GTAGAAGGTCACGTCA CAAC	CCTAACACTGTCTGGTAA
*Acan*	CTAGCTGCTTAGCAGGG ATAACG	GATGACCCGCAGAGTCAC AAAG
*Sox9*	AGGAAGCTGGCAGACC AGTACC	GGGTCTCTTCTCGCTCTC GTTCA
*Col2a1*	CTGGTGGAGCAGCAA GAGC	GTGGACAGTAGACGGAG GAAAG
*ADAMT4*	CTCCTGCCTTTAGCC TGGTC	CCCAAAGGCTGGTAATC GGT
*MMP3*	TGATGGGCCTGGAAT GGTC	TTCATGAGCAGCAACCAG GAATAG
*MMP13*	TGATGATGAAACCTGG ACAAGCA	GAACGTCATCATCTGGG AGCA

### Western Blotting

Protein was extracted from tissue and cell samples in radioimmunoprecipitation assay buffer supplemented with a proteasome inhibitor (Beyotime, China), and was separated by sodium dodecyl sulfate-polyacrylamide gel electrophoresis. Next, the proteins were transferred to a polyvinylidene fluoride membrane (Millipore, Shanghai, China) by electroblotting. The membranes were blocked with 5% non-fat milk for 2 h and incubated overnight with antibodies against cleaved caspase-1 (ab207802, Abcam, Shanghai, China), NT-GSDMD (ab215203, Abcam), IL-1β (12703, Cell Signaling Technology, Shanghai, China), IL-18 (54943, Abcam), TXNIP (ab188865, Abcam), or NLRP3 (ab263899, Abcam). GAPDH (ab181602, Abcam) was used as the internal control. After washing three times with Tris-buffered saline containing 0.1% Tween-20 (TBST), the membranes were incubated with the horseradish peroxidase-conjugated secondary antibodies (Beyotime) for 1 h at room temperature. Immunoreactive bands were visualized using an enhanced chemiluminescence substrate (Millipore). Signal intensity was measured using the Bio-Rad XRS chemiluminescence detection system (Bio-Rad laboratories, Hercules, CA, United States).

### Microarray Analysis

Total RNA was extracted from tissues using TRIzol reagent (Takara) and quantified using a NanoDrop ND-2000 (Thermo Fisher Scientific, Shanghai, China). After purified with a QIAGEN RNeasy Kit (QIAGEN, Shanghai, China), total RNA was amplified and labeled with Cy-3. Next, RNA was hybridized for 17 h at 65°C and washed with Gene Expression Wash Buffers 1 and 2 (Agilent Technologies, Beijing, China). Array images were acquired using Agilent Scanner G5761A (Agilent Technologies) and analyzed using Agilent Feature Extraction software (version 12.0.1.1). Quantile normalization and subsequent data processing were performed by using the GeneSpring v14.8 software package (Agilent Technologies). Next, miRNAs with at least three out of the six samples having flags in Detected were chosen for further analysis. Significantly differentially expressed miRNAs (*p* < 0.01) were identified and subjected to hierarchical cluster analysis. Heat map and gene ontology (GO) enrichment analyses were performed to identify the biological functions of the differentially expressed miRNAs.

### Immunofluorescence Staining

Nucleus pulposus cells were cultured in 12-well plates. After fixation with 4% paraformaldehyde for 10 min at room temperature, the cells were treated with 0.1% Triton X-100 for 10 min and incubated with 2% bovine serum albumin for 30 min at room temperature. Next, cells were incubated with anti-collagen II (ab34712, Abcam) and anti-aggrecan antibodies (ab3773, Abcam) overnight and then incubated with an Alexa Fluor 555-labeled secondary antibody for 1 h at room temperature in the dark. Nuclei were stained with 4′,6- diamidino-2-phenylindole (DAPI, Sigma-Aldrich, Shanghai, China) for 5 min. Fluorescence was detected using a fluorescence microscope (DM5500; Leica, Wetzlar, Germany).

### Alcian Blue Staining

Nucleus pulposus cells were cultured in six–well plates for 7 days. The cells were fixed with 4% paraformaldehyde for 10 min and incubated in Alcian blue staining solution (Sigma–Aldrich) for 30 min at room temperature. After washing three times with distilled water, three fields per well were chosen randomly for observation under an inverted microscope (Leica).

### Detection of Cellular Reactive Oxygen Species (ROS)

Nucleus pulposus cells were cultured in 12–well plates. After treatment with acid or N–acetyl cysteine (NAC, Sigma–Aldrich), the NPCs were rinsed and incubated with 5μM DCFH–DA (Sigma–Aldrich) in the dark at 37°C for 30 min. Fluorescence was detected using a fluorescence microscope (DM5500; Leica).

### Flow Cytometry

Flow cytometry was performed to assess pyroptotic cell death. Fluorochrome inhibitor of caspase–1 (caspase–1 FLICA) and propidium iodide (PI) were added to NPCs, and pyroptotic death was assessed by flow cytometry after FLICA/PI staining. PI (+) and caspase–1 FLICA (+) was defined as pyroptosis.

### Animal and Surgical Procedure

Male Sprague Dawley rats weighing 250 g were obtained from the Animal Center of the Academy of Medical Science of Zhejiang Province. All procedures in this study were approved by the Institutional Animal Care and Use Committee of Zhejiang University. Rats were anesthetized with 1% sodium pentobarbital (Sigma–Aldrich) and a 20 G sterile needle was inserted into the disk of coccygeal vertebrae (Co)7/Co8 and Co8/Co9 to a depth of approximately 5 mm. Next, the needle was rotated 360° and maintained for 30 s before being removed (Masuda et al., 2005). For NP generation and injection, 3 μg of miR–141 mimic or inhibitor was packed in a NP carrier system (MaxSuppressor *in vivo* RNALANCEr II Kit and Lipid Extruder, BIOO Scientific, Beijing, China). Two weeks after the surgical procedure, 32 rats were randomized into four groups: the normal group (without needle puncture and other treatments); the NC group (with needle puncture and phosphate buffer solution injection); the miR–141 mimic group (with needle puncture and miR–141 mimic injection); and the miR–141 inhibitor group (with needle puncture and miR–141 inhibitor injection). The injection was performed using a microsyringe with a 31–G needle and 3 μL of liquid. miR–141 mimic or inhibitor was injected every week.

### Histological, Immunohistochemical, and Biochemical Analysis

Samples of rat tails were harvested at 16 weeks after injection. The samples were fixed in 4% paraformaldehyde for 3 days before being immersed in decalcifying solution. The samples were dehydrated, embedded in paraffin, and sectioned at 3.5 μm thickness using a microtome. For histological analysis, haematoxylin and eosin (H&E) and safranine O–fast green staining were performed separately on consecutive tissue sections. Cellularity and morphology were assessed using a previously described grading scale and presented as a histological score (Han et al., 2008). For immunohistochemical analysis, tissue sections were treated with 3% H_2_O_2_ for 10 min and blocked with goat serum for 30 min at room temperature. The tissue sections were incubated with anti–collagen II antibody (Abcam) overnight at 4°C, then with a biotin–conjugated secondary antibody for 1 h at room temperature and detected by the SABC method. Images were obtained using a microscope (Leica). For biochemical analysis, NP samples of rats were frozen at −80°C and lyophilized for 24 h, and the dry weight was recorded. The glycosaminoglycan (GAG) content was determined by Blyscan assay (Biocolor, Beijing, China) according to the manufacturer’s protocols and normalized to the dry weight.

### Statistical Analysis

All experiments were repeated three times and data are presented as the means ± standard deviation. A two–tailed Student’s *t*–test and one–way analysis of variance followed by Tukey *post hoc* test were performed to assess the significance of differences using SPSS ver. 22.0 (IBM Corp., Armonk, NY, United States). A value of *p* < 0.05 was considered to indicate statistical significance.

## Results

### Pyroptosis Was Related With Disk Degeneration

**FIGURE 1:**
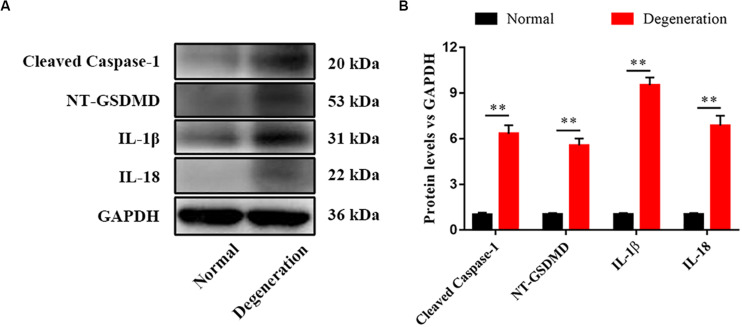
Pyroptosis was related with disk degeneration. **(A)** Protein expression levels of Cleaved caspase–1, NT–GSDMD, IL–1β, and IL–18 in the nucleus pulposus (NP) of healthy donors and IDD patients were measured by western blotting analysis. **(B)** Quantification of the protein expression levels in each group. Data represent mean ± SD; ^∗∗^*p* < 0.01.

We assessed pyroptosis in normal and degenerative NP by western blotting. The protein levels of cleaved caspase–1, NT–GSDMD, IL–1β, and IL–18 in the degenerative NP were significantly higher (6. 32–, 5. 56–, 9. 52–, and 6.86–fold) than those in the normal NP (Figures 1A,B).

### miR-141 Was Highly Expressed in Degenerated NP

**FIGURE 2:**
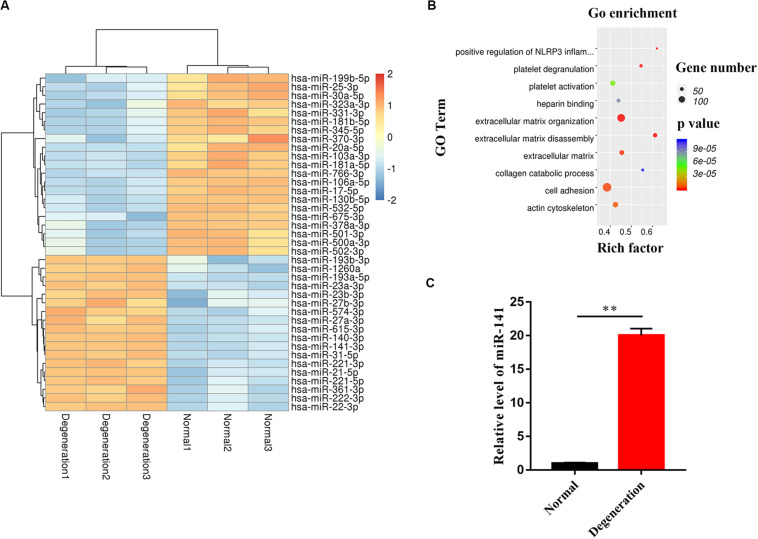
miR-141 was highly expressed in degenerated nucleus pulposus (NP). **(A)** The heatmap showed differentially expressed MicroRNAs (miRNAs) between normal and degenerated NP. **(B)** Gene ontology (GO) terms with significant *p* values for biological processes, molecular function, cellular component. **(C)** The expression level of miR-141 in normal and degenerated NP was verified by polymerase chain reaction (PCR) and normalized to U6. Data represent mean ± SEM; ^∗∗^*p* < 0.01.

We performed miRNA microarray analysis to identify differentially expressed miRNAs between normal and degenerative NP. Only miRNAs with a mean fold change > 5 or < 0.2 and a *p*-value of < 0.01 were selected for further analysis. Thirty-nine miRNAs were significantly dysregulated; of them, miR-141 was the most significantly upregulated miRNA in the degenerative group compared to the normal group (Figure 2A). Dysregulated mRNAs were also subjected to GO analysis. The results showed that the GO terms with the most significant *p* values for biological processes, molecular function, and cellular component were related to ECM disassembly (GO:0022617), ECM organization (GO:0030198), platelet degranulation (GO:0002576), and positive regulation of NLRP3 inflammasome complex assembly (GO:1900227) which was involved in the process of pyroptosis (Figure 2B). RT-qPCR showed that the expression level of miR-141 in the degeneration group was almost 20.05-fold that in the normal group (Figure 2C).

### miR-141 Induced Pyroptosis in NPCs

**FIGURE 3:**
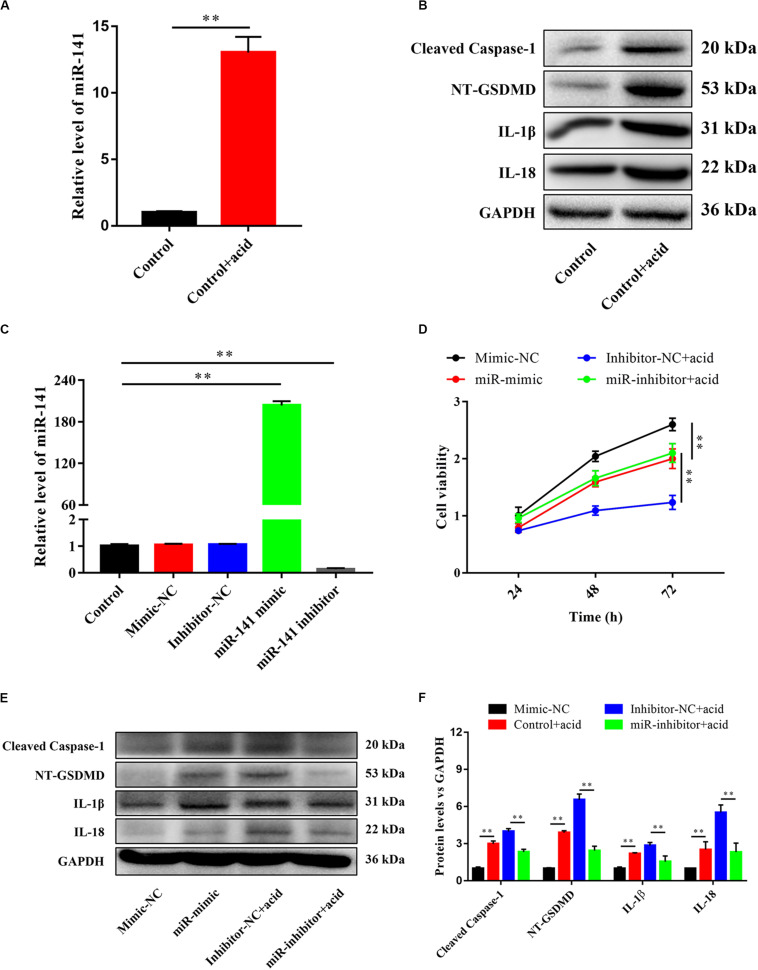
miR-141 induced pyroptosis in nucleus pulposus cells (NPCs). **(A)** The expression level of miR-141 in the control and control + acid groups. **(B)** Protein expression levels of Cleaved caspase-1, NT-GSDMD, IL-1β, and IL-18 in the control and control + acid groups were measured. **(C)** NPCs were transfected with miR-NC, miR-141 mimic, and miR-141 inhibitor. The expression levels of miR-141 was measured by polymerase chain reaction (PCR) and normalized to U6. **(D)** Cell viability after transfection was measured by Cell Counting Kit-8 (CCK8). **(E)** Protein expression levels of Cleaved caspase-1, NT-GSDMD, IL-1β, and IL-18 in each group on day 3 were measured. **(F)** Quantification of the protein expression levels in each group. Data represent mean ± SEM; ^∗∗^*p* < 0.01.

An acidic environment significantly increased the expression of miR-141 (Figure 3A) and the protein levels of cleaved caspase-1, NT-GSDMD, IL-1β, and IL-18 (Figure 3B). The expression level of miR-141 was quantified after the transfection of mimic-NC, inhibitor-NC, miR-141 mimic, and miR-141 inhibitor. No significant difference was observed between the control mimic-NC, and inhibitor-NC groups. The expression levels of miR-141 in the miR-141 mimic and miR-141 inhibitor groups were 203.71- and 0.14-fold that in the control group (Figure 3C). The viability of NPCs in the miR-mimic group was lower than that in the mimic-NC group at each time point. The miR-inhibitor group showed higher cell viability compared to the inhibitor-NC group in an acidic environment, particularly at 72 h (Figure 3D). We also assessed pyroptosis in NPCs by measuring the protein levels of cleaved caspase-1, NT-GSDMD, IL-1β, and IL-18. The miR-141 mimic significantly promoted pyroptosis by increasing the levels of cleaved caspase-1, NT-GSDMD, IL-1β, and IL-18; the miR-141 inhibitor prevented pyroptosis by downregulating cleaved caspase-1, NT-GSDMD, IL-1β, and IL-18 compared with the inhibitor-NC group (Figures 3E,F).

### miR-141 Suppressed the Matrix Synthesis Function of NPCs

**FIGURE 4:**
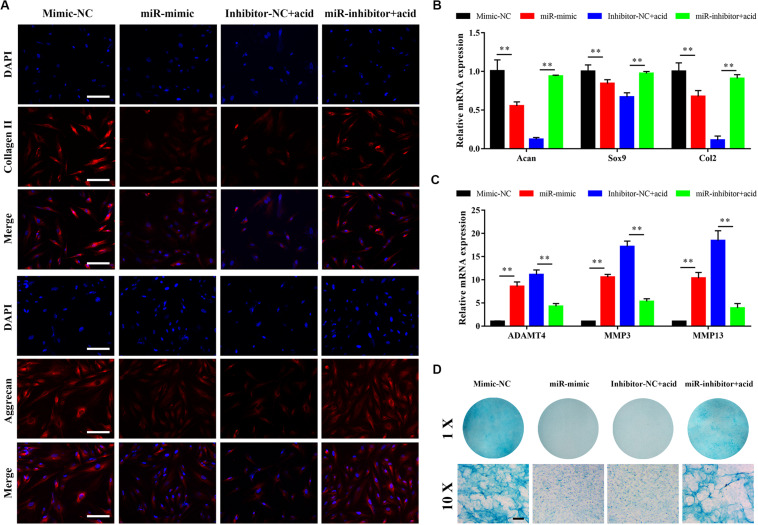
miR-141 suppressed the matrix synthesis function of nucleus pulposus cells (NPCs). **(A)** Immunofluorescence staining showed the protein expression levels of collagen (red) and aggrecan (red) on day 7. The nuclei were stained with 4′,6-diamidino-2-phenylindole (DAPI). **(B)** The gene expression level of Acan, Sox9, and Col2 of NPCs in different groups were measured by polymerase chain reaction (PCR) and normalized to 18 s. **(C)** The gene expression level of ADAMT5, matrix metallopeptidase (MMP)3, and MMP13 of NPCs in different groups were measured by PCR and normalized to 18 s. **(D)** sGAG synthesized by NPCs was observed by alcian blue staining on day 7. Data represent mean ± SEM; ^∗∗^*p* < 0.01. Scale bar = 200 μm.

The effect of miR-141 on matrix synthesis by NPCs was evaluated. The synthesis of collagen II and aggrecan was downregulated by miR-141. The synthesis of collagen II and aggrecan was increased by miR-141 inhibition (Figure 4A). The expression levels of *acan*, *sox9*, and *col2* in the miR-mimic group were significantly lower than those in the mimic-NC group. The miR-inhibitor + acid group had higher expression levels of these genes than the inhibitor-NC + acid group (Figure 4B). Also, the expression of *ADAMT4*, *MMP3*, and *MMP13* was increased in the miR-mimic group and decreased in the miR-inhibitor group compared with the respective NC groups (Figure 4C). Alcian blue staining indicated a lower GAG content in the miR-mimic group than that in the mimic-NC group. An acidic environment inhibited GAG deposition; the GAG content in the miR-inhibitor group was higher than that in the inhibitor-NC group (Figure 4D).

### miR-141 Modulated Pyroptosis in NPCs by Targeting ROS and TXNIP/NLRP3 Pathway

**FIGURE 5:**
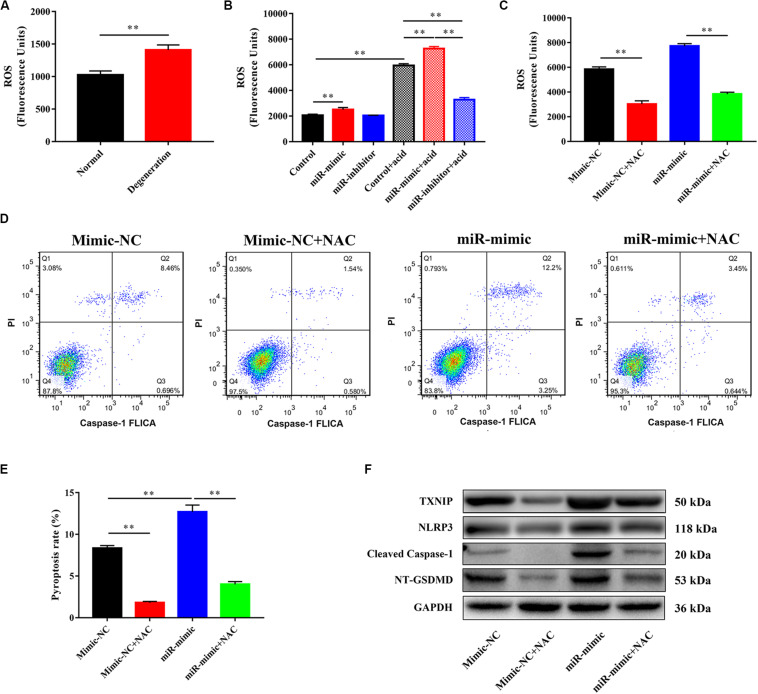
miR-141 modulated pyroptosis in nucleus pulposus cells (NPCs) by targeting reactive oxygen species (ROS) and TXNIP/NLRP3 pathway. **(A)** The level of ROS in normal and degenerated human nucleus pulposus (NPs). **(B)** Measurement of intracellular ROS generation in each group using a DCFH-DA probe by fluorometry. **(C)** N-acetyl cysteine (NAC) was used to decrease the ROS generation, and intracellular ROS generation in each group after using NAC was measured. **(D)** Pyroptosis in each group was assessed using FLICA/PI staining and flow cytometric analysis. **(E)** Quantification of pyroptosis rate in each group. **(F)** Protein expression levels of Cleaved caspase-1, NT-GSDMD, IL-1β, and IL-18 in each group after using NAC were measured. Data represent mean ± SEM; ^∗∗^*p* < 0.01.

We investigated the mechanism underlying miR-141-induced pyroptosis. The reactive oxygen species (ROS) level in the degeneration group was higher than that in the normal group (Figure 5A). miR-141 significantly increased intracellular ROS generation with and without acid compared with the control group. However, the miR-141 inhibitor decreased ROS generation by NPCs in an acidic environment (Figure 5B). NAC decreased ROS generation by NPCs in the mimic-NC and miR-mimic groups (Figure 5C). Furthermore, the pyroptosis rate and caspase-1 activity in the miR-mimic group were markedly higher than those in the mimic-NC group. NAC downregulated the pyroptosis rate in the mimic-NC + NAC and miR-mimic + NAC groups compared with the mimic-NC and miR-mimic groups, respectively (Figures 5D,E). The miR-mimic group also showed increased protein levels of TXNIP, NLRP3, cleaved caspase-1, and NT-GSDMD compared with the mimic-NC group. After the addition of NAC, the mimic-NC and miR-mimic groups showed decreased protein levels of TXNIP, NLRP3, cleaved caspase-1, and NT-GSDMD (Figure 5F).

### An miR-141 Inhibitor Prevented the Degeneration of IVDs

**FIGURE 6:**
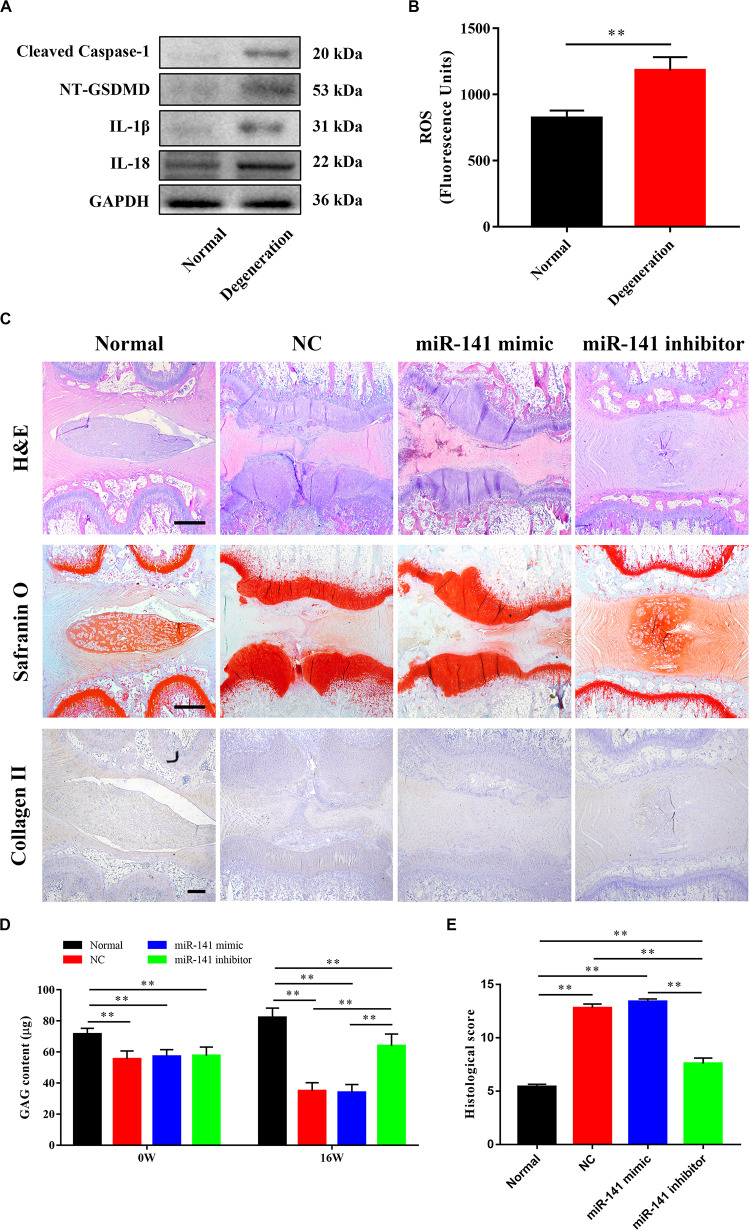
miR-131 inhibitor prevented the degeneration of intervertebral disks (IVDs). **(A)** The pyroptosis in the degenerated nucleus pulposus (NPs) of rat model was measured by western blotting analysis. **(B)** The level of ROS in normal and degenerated rat NPs. **(C)** Representative haematoxylin and eosin (H&E) and Safranin O staining, and immunohistochemical detection of collagen II of disks from different groups were observed. All samples were harvested at 16 weeks after injection. **(D)** The content of sGAG in each group at 0 and 16 weeks after injection were quantified by Blyscan assay. **(E)** Histological grade of each group was evaluated at 16 weeks after injection. Data represent mean ± SEM; ^∗∗^*p* < 0.01. Scale bar = 500 μm.

The ability of miR-141 to prevent IVD degeneration was verified *in vivo*. We first assayed pyroptosis in the rat disk degeneration model. The protein levels of cleaved caspase-1, NT-GSDMD, IL-1β, and IL-18 in the degeneration group were significantly higher than those in the normal group (Figure 6A). Also, the degeneration group had a higher ROS level than the normal group (Figure 6B). H&E staining showed that the lamellar sheets of the AF in the normal group were regular and that the NP was well organized with cells and ECM. No regular structure of the AF and NP was observed in the NC and miR-141 mimic groups. The NP in the miR-141 inhibitor group was more regular and well organized than that in the NC group. Safranin O staining, indicating GAG deposition, was stronger in the control and miR-141 inhibitor groups than those in the NC and miR-141 mimic groups. Immunohistochemical staining showed the presence of collagen II in the NP of only the normal and miR-141 inhibitor groups. Few areas of the NP were immune-positive for collagen II in the NC and miR-141 mimic groups (Figure 6C). The GAG contents at weeks 0 and 16 in the normal group were significantly higher than those in the other groups. The GAG contents in the NC, miR-141 mimic, and miR-141 inhibitor groups were similar at week 0. However, the miR-141 inhibitor group showed a higher GAG content than the NC and miR-141 mimic groups at week 16 (Figure 6D). Histological scoring was performed to evaluate the degeneration of IVDs. The score of the control was 5.4 ± 0.55, significantly lower than those in the other groups. The miR-141 mimic group had the highest score (13.4 ± 0.55). The histological score of the miR-141 inhibitor group (7.6 ± 1.14) was lower than that of the NC group (12.8 ± 0.84; Figure 6E).

## Discussion

Nucleus pulposus degeneration, which is caused by disruption of ECM anabolism and catabolism of NPCs, is the trigger for IVD degeneration (Fontana et al., 2015). Pyroptosis is a form of programed cell death and is accompanied by an inflammatory response (Hu et al., 2018). It plays an important role in regulating cell viability and mediating ECM deposition (Zhaolin et al., 2019). However, the role of pyroptosis in IVD degeneration is unclear. We hypothesize that pyroptosis occurs during IVD degeneration and modulates the viability of, and ECM synthesis by, NPCs. miRNAs play critical roles in cell proliferation, function, and pyroptosis. We investigated the relationship between NP degeneration, pyroptosis, and miRNA. We also investigated the mechanism underlying the miRNA-mediated regulation of pyroptosis in NPCs.

In some cases, the pyroptosis pathway can trigger apoptosis. The relationship between apoptosis and pyroptosis in NPCs is unclear (Green, 2019). However, pyroptosis and apoptosis co-exist in cardiomyocytes (Fisch et al., 2019; Li et al., 2019). Therefore, we hypothesize that within the same patient, a portion of NPCs undergo apoptosis and the remainder experience pyroptosis. The proportions of NPCs that undergo pyroptosis may depend on the inducing factor and the phase of degeneration, and a molecular switch may control the initiation of pyroptosis and apoptosis (Lee et al., 2018; Fritsch et al., 2019). Pyroptosis is involved in the degeneration of diverse tissues. Age-related macular degeneration is regulated by the activation of NLRP3 inflammasome signaling (Sun et al., 2020). Pyroptosis of chondrocytes is also involved in cartilage degeneration (Hu et al., 2020). However, no study has assessed the relationship between pyroptosis and IVD degeneration. We evaluated pyroptosis in degenerative NP and found that the levels of markers of pyroptosis (such as cleaved caspase-1, NT-GSDMD, IL-1β, and IL-18) were significantly increased in degenerative NP compared to normal NP. Therefore, pyroptosis is involved in the degeneration of IVDs. A low pH increased the levels of cleaved caspase-1, NT-GSDMD, IL-1β, and IL-18. Therefore, the acidic microenvironment of degenerative IVD may trigger pyroptosis of NPCs.

We next identified differentially expressed miRNAs between normal and degenerative NP by microarray analysis. The results are consistent with previous studies. For example, miR-222 and miR-21 are reportedly upregulated in degenerative NPCs; this is in agreement with our findings (Wang W.J. et al., 2018; Zhang et al., 2019b). Wang W. et al. (2018) reported that miR-199 is downregulated in degenerated NPCs, also in agreement with our results. The expression of miR-17 in degenerative NPCs was low, which was linked to a higher IVD degeneration grade during inhibition (Song J. et al., 2018). However, the expression levels of some miRNAs reported to be involved in regulating IVD were not significantly different, such as miRNA-124 and miRNA-143 (Wang X.Q. et al., 2019; Yang et al., 2019). This discrepancy may be a result of the source of samples and the systemic error of microarray analysis. miR-141 was significantly upregulated in the degenerative group compared with the normal group by microarray and PCR. miR-141 reportedly regulates apoptosis of NPCs (Ji et al., 2018). Apoptosis of different types of cancer cells mediated by miR-141 has also been reported (Wang N. et al., 2018; Zhang et al., 2019a). The levels of pyroptosis markers were upregulated by an miR-141 mimic and downregulated by an miR-141 inhibitor; by contrast, cell viability was downregulated by an miR-141 mimic and upregulated by an miR-141 inhibitor. These results implicate miR-141 in pyroptosis of NPCs.

We further investigated the mechanism by which miR-141 regulates pyroptosis. The acidic microenvironment in degenerative IVD induces excessive ROS generation (Gonzalez et al., 2020). Indeed, the ROS levels were higher in degenerated NPs compared to normal NPs from human and rat. Also, miRNA-141 increased ROS generation in NPCs and its effect was synergistic with that of acidity. ROS activates TXNIP/NLRPS signaling, which plays an important role in pyroptosis (Chavarria-Smith and Vance, 2015; Gu et al., 2019). In this study, miRNA-141 upregulated the expression of TXNIP and NLRP3 in a ROS-dependent manner. Therefore, miRNA-141 induced pyroptosis in NPCs via the ROS/TXNIP/NLRPS axis. However, the mechanism by which miRNA-141 induced ROS generation warrants further investigation.

Extracellular matrix synthesis by NPCs was also modulated by miRNA-141. *Acan*, *Sox9*, *Col2*, *MMP3*, *MMP13*, and *ADAMT4* reflect the balance between ECM catabolism and anabolism by NPCs (Zhou et al., 2015). The expression levels of *Acan*, *Sox9*, and *Col2* were downregulated by the miRNA-141 mimic and upregulated by the miRNA-141 inhibitor. By contrast, the expression levels of *MMP3*, *MMP13*, and *ADAMT4* were upregulated by the miRNA-141 mimic and downregulated by the miRNA-141 inhibitor. The protein levels of collagen II and aggrecan, and GAG synthesis by NPCs indicated that miRNA-141 inhibited ECM anabolism and aggravated its catabolism by NPCs (Wang X. et al., 2018; Wang Y. et al., 2019). The NF-κB signaling pathway is a key regulator of ECM deposition; its activation upregulates matrix metallopeptidase (MMP)3 and MMP13 expression and downregulates that of collagen II in chondrocytes (Hu et al., 2020). miRNA-141 increased the generation of ROS, which activate the NF-κB signaling pathway, and increased the expression of MMPs, inducing ECM degradation (Wang X. et al., 2018; Wang Y. et al., 2019). miRNA-141 itself is a positive regulator of NF-κB signaling pathway (Ji et al., 2018). Therefore, NF-κB signaling regulated by miRNA-141 may be responsible for ECM catabolism by NPCs.

*In vivo*, pyroptosis occurred in the rat disk degeneration model. The degenerative IVD had an abnormal NP structure and decreased GAG and collagen II contents. The miRNA-141 mimic aggravated IVD degeneration, which also demonstrated the negative regulator of miRNA-141 in NPCs. The miRNA-141 inhibitor group had NP of a more regular shape and higher collagen II and GAG contents. However, the differences between normal and miRNA-141 inhibitor-treated IVD indicated that miRNA-141 inhibitors cannot regenerate degenerated IVD. IVD degeneration is modulated by nutrition, mechanical loading, and genetics (Urban and Roberts, 2003; Urban et al., 2004; Navone et al., 2018). The miRNA-141 inhibitor suppressed pyroptosis, upregulated ECM synthesis by NPCs, and prevented IVD degeneration. Therefore, inhibition of pyroptosis may contribute to IVD degeneration treatment, but other modalities are needed.

## Conclusion

miRNA-141 induced pyroptosis and ECM catabolism of NPCs. miRNA-141 increased ROS generation in NPCs and activated TXNIP/NLRP3 signaling, leading to pyroptosis. Also, miR-141 inhibitor prevented IVD degeneration *in vivo*. Our work will attract attention to pyroptosis in IVD and facilitate the discovery of more efficacious treatments for IVD degeneration.

## Data Availability Statement

The original contributions presented in the study are included in the article/supplementary material, further inquiries can be directed to the corresponding authors.

## Ethics Statement

The animal study was reviewed and approved by Ethics Committee of The Second Affiliated Hospital of Zhejiang University School of Medicine. Written informed consent was obtained from the individual(s) for the publication of any potentially identifiable images or data included in this article.

## Author Contributions

QX, WC, and NZ designed the research. QX performed the research, analyzed the data, and wrote the manuscript. HX performed the research and analyzed the data. JW performed the research. All authors contributed to the article and approved the submitted version.

## Conflict of Interest

The authors declare that the research was conducted in the absence of any commercial or financial relationships that could be construed as a potential conflict of interest.
